# Vasopressin Cells in the Rodent Olfactory Bulb Resemble Non-Bursting Superficial Tufted Cells and Are Primarily Inhibited upon Olfactory Nerve Stimulation

**DOI:** 10.1523/ENEURO.0431-18.2019

**Published:** 2019-07-09

**Authors:** Michael Lukas, Hajime Suyama, Veronica Egger

**Affiliations:** Institute of Zoology, Neurophysiology, University of Regensburg, 93040 Regensburg, Germany

**Keywords:** calcium imaging, electrophysiology, olfactory bulb, social, tufted cells, vasopressin

## Abstract

The intrinsic vasopressin system of the olfactory bulb is involved in social odor processing and consists of glutamatergic vasopressin cells (VPCs) located at the medial border of the glomerular layer. To characterize VPCs in detail, we combined various electrophysiological, neuroanatomical, and two-photon Ca^2+^ imaging techniques in acute bulb slices from juvenile transgenic rats with eGFP-labeled VPCs. VPCs showed regular non-bursting firing patterns, and displayed slower membrane time constants and higher input resistances versus other glutamatergic tufted cell types. VPC axons spread deeply into the external plexiform and superficial granule cell layer (GCL). Axonal projections fell into two subclasses, with either denser local columnar collaterals or longer-ranging single projections running laterally within the internal plexiform layer and deeper within the granule cell layer. VPCs always featured lateral dendrites and a tortuous apical dendrite that innervated a single glomerulus with a homogenously branching tuft. These tufts lacked Ca^2+^ transients in response to single somatically-evoked action potentials and showed a moderate Ca^2+^ increase upon prolonged action potential trains.

Notably, electrical olfactory nerve stimulation did not result in synaptic excitation of VPCs, but triggered substantial GABA_A_ receptor-mediated IPSPs that masked excitatory barrages with yet longer latency. Exogenous vasopressin application reduced those IPSPs, as well as olfactory nerve-evoked EPSPs recorded from external tufted cells. In summary, VPCs can be classified as non-bursting, vertical superficial tufted cells. Moreover, our findings imply that sensory input alone cannot trigger excitation of VPCs, arguing for specific additional pathways for excitation or disinhibition in social contexts.

## Significance Statement

Efficient sensing of conspecific odor signatures is essential for most rodent social behavior. Although olfactory bulb vasopressin was shown to be a potent facilitator of social odor processing, little is known on the cellular substrate of the intrinsic vasopressin system. Here we provide a detailed characterization of the anatomic and electrophysiological properties of the bulbar vasopressin cells. Although we also identify several targets of vasopressin action, we find that stimulation of the sensory inputs to the bulb results primarily in vasopressin cell inhibition, implying that excitation of the bulbar vasopressin system requires additional still unknown excitatory or dis-inhibitory inputs, which might confer social specificity. These insights may complement the knowledge on vasopressinergic modulation of social stimuli in limbic brain structures.

## Introduction

The neuropeptide vasopressin (VP) is primarily synthesized in neurons located within the supraoptic, paraventricular, and suprachiasmatic nuclei of the hypothalamus (Ludwig and Leng, 2006). These neurons release VP from their axonal projections to the neurohypophysis into the bloodstream to exert its peripheral physiologic functions as a neurohormone, e.g., water retention in the kidney (Ondrasek, 2016). In the CNS, VP is known as a key modulator of social behavior and cognition in mammals, including rodents and humans (Meyer-Lindenberg et al., 2011; Lukas and Neumann, 2013; Lukas and de Jong, 2016). In this context, relevant VP release was shown to occur from somata and dendrites of VP cells (VPCs) in the hypothalamus as well as from hypothalamic and extra-hypothalamic fibers that target the components of the social behavior network throughout the mammalian brain, e.g., the lateral septum, the medial extended amygdala, the anterior and ventromedial hypothalamus, and the periaqueductal gray (Sterba, 1974; Buijs et al., 1983; Ondrasek, 2016). The extra-hypothalamic brain regions that also synthesize and release VP during social interactions are the bed nucleus of stria terminalis, the medial amygdala, and the olfactory bulb (OB), i.e., the first center of olfactory processing (De Vries and Buijs, 1983; Tobin et al., 2010; Lukas and de Jong, 2016).

Olfactory processing is an essential component of mammalian social communication, in rodents, sheep, and even humans (Porter et al., 1986; Brennan and Kendrick, 2006). Especially in rodents, the olfactory system is regarded as the main sensory pathway for mediating recognition and discrimination of individual con-specifics (Camats Perna and Engelmann, 2017). Several pharmacological studies suggest that endogenous VP release within the OB facilitates the discrimination of known and new individuals via their odor signatures (Dluzen et al., 1998a,b; Tobin et al., 2010). The source of this VP release are bulbar VPCs, a subpopulation of glutamatergic tufted cells with lateral dendrites (Macrides and Schneider, 1982; Hamilton et al., 2005), that reside at the border between the glomerular layer (GL) and the external plexiform layer (EPL) in both the accessory OB (AOB) and the main OB (MOB; Tobin et al., 2010; Wacker et al., 2011). The presence of VPCs in both pathways for odorant detection (volatile/MOB and non-volatile/AOB) is in line with the view that volatile odor signals are especially important for the coding of individual body odor signatures (Brennan and Kendrick, 2006) and thus the AOB and MOB play complementary roles in processing social odor recognition (Baum and Kelliher, 2009; Stowers and Kuo, 2016).

As mentioned in the previous paragraph, VP enhances social recognition of individuals on the level of the OB, but what could be the cellular mechanisms that are responsible for this facilitation of social odor processing? As a first step toward resolving these questions, here we provide a detailed investigation of basic electrophysiological and neuroanatomical properties of the OB VPCs, including their axonal projections. We also set out to identify synaptic inputs to VPCs, which turns out to be a challenging task because here we observe that they receive mostly inhibition on stimulation of olfactory sensory axons. Moreover, we investigate the expression of VP in VPC axons and dendrites including their elaborate glomerular apical tuft, and test for effects of VP on glomerular synaptic signaling. To further explore potential mechanisms of dendritic release within a glomerulus, we also characterize the excitability of the apical dendritic tuft. Our results imply that bulbar VPCs are likely to be involved in a broad range of complex interactions both within glomeruli and deeper layers of the bulb.

## Materials and Methods

### Experimental animals

All experiments were conducted according to national and institutional guidelines, the rules laid down by the EC Council Directive (86/89/ECC) and German animal welfare. Wistar rats of either sex were either purchased from Charles River Laboratories or bred onsite in the animal facilities at the University of Regensburg. Heterozygous VP-eGFP Wistar rats (Ueta et al., 2005) of either sex that were used to identify VPCs in electrophysiological and imaging experiments were all bred at the University of Regensburg.

### Slice preparation

Rats (postnatal day 11–21) were deeply anaesthetized with isoflurane and decapitated. Horizontal OB slices (300 µm) were cut in ice-cold carbogenized [O_2_ (95%), CO_2_ (5%)] artificial extracellular fluid [ACSF; (in mm): 125 NaCl, 26 NaHCO_3_, 1.25 NaH_2_PO_4_, 20 glucose, 2.5 KCl, 1 MgCl_2_, and 2 CaCl_2_] using a vibratome (Vibracut, Leica Biosystems) followed by incubation in carbogenized ACSF for 30 min at 36°C and then kept at room temperature (∼21°C) until experimentation.

### Electrophysiology

External tufted cells (eTCs), mitral cells (MCs), and middle tufted cells (mTCs) were identified by their morphological appearance and their localization in the clearly defined GL, MC layer (MCL), and EPL, respectively (Halász 1990). VPCs were identified in OB slices from VP-eGFP rats excited with LED illumination (470 nm nominal wavelength, M470L2, Thorlabs) under a modified Zeiss Axioplan microscope (Carl Zeiss Microscopy). Epifluorescence was filtered by a long-pass dichroic mirror (490 nm cutoff, DMLP490R, Thorlabs) and an emission filter (510 ± 21 nm, MF510-42, Thorlabs) and visualized with a digital camera (VisiCAM-100, Visitron Systems). To perform somatic whole-cell patch-clamp recordings cells were visualized by infrared gradient-contrast illumination via an IR filter (Hoya) and patched with pipettes sized 4–6 MΩ. Recordings were performed with an EPC-10 (HEKA). Series resistances measured 10–30MΩ. The intracellular solution contained the following (in mm): 130 K-methylsulfate, 10 HEPES, 4 MgCl_2_, 4 Na_2_ATP, 0.4 NaGTP, 10 NaPhosphocreatine, 2 ascorbate, pH 7.2. The ACSF was gassed with carbogen and contained (in mm): 125 NaCl, 26 NaHCO_3_, 1.25 NaH_2_PO_4_, 20 glucose, 2.5 KCl, 1 MgCl_2_, and 2 CaCl_2_. Experiments were performed at room temperature (∼21° C). The average resting potential of MCs/mTCs and eTCs/VPC was ranging from −60 to −75 mV and −55 to −60 mV, respectively, similar to previous data (Heyward et al., 2001; Hayar et al., 2004b; Tobin et al., 2010). Leaky cells with a holding current above ∼−30 pA were rejected. Experiments that showed a substantial drift in resting *V*_m_ were rejected.

Spontaneous activity (i.e., IPSPs in VPCs and bursts in eTCs) was recorded in current-clamp mode at resting V_m_. To characterize the firing pattern and passive properties of VPCs, eTCs and other tufted OB cell types, including membrane time constant (τ_m_), input resistance (R_i_), firing threshold, first/last spike amplitude ratio, first/last afterhyperpolarization (AHP) ratio, sag amplitude relative to the hyperpolarization level at the end of the current step, rebound amplitude, and coefficient of variance (CV) of the interspike interval (ISI), polarizing step pulses were applied via the patch pipette for 600–800 ms each. Firing pattern analysis was performed using Origin 2017 (OriginLab).

### Olfactory nerve stimulation

Olfactory nerve (ON) stimulation was performed with a custom-built four-channel electrode (Chatterjee et al., 2016; Lukas et al., 2018). Briefly, the four electrodes consisted of Teflon-coated silver wires (diameter uncoated 75 µm, coated 140 µm, item AG-3T, Science Products). The electrode was connected to a 4-channel stimulator (STG 1004, Multi-Channel Systems) that is controlled from a PC via an USB connection. In current mode, the maximal stimulation strength per channel is 800 µA. The grounds from the stimulator channels were connected to a common wire and then to mass. The four-channel electrode was lowered on top of the acute brain slice under visual control using a manual manipulator (LBM-7, Scientifica). During ON stimulation only the channel eliciting the best signal was used to stimulate the ON. The stimulation strength was adjusted via the stimulator’s software (MC_Stimulus, V 2.1.5); the output of the stimulator was triggered via a TTL signal from the electrophysiology software (Patchmaster, HEKA). Stimulation strengths sufficient to elicit MC, eTC, and VPC responses were mostly in the range of 50–400 µA and 300–500 µA for 100 µs, respectively.

### Pharmacology

The pharmacological agents used during electrophysiological experiments include 1(S),9(R)-(-)-Bicuculline methbromide (50 µm; Sigma-Aldrich), [Arg^8^]-vasopressin acetate salt (1 µm; Sigma-Aldrich), and the Manning compound, a selective VP 1a/oxytocin receptor antagonist [10µm; d(CH_2_)_5_[Tyr(Me)^2^]AVP; Kruszynski et al., 1980]. The Manning compound was generously provided by Dr. Maurice Manning (University of Toledo, Toledo, OH).

### Ca^2+^ imaging

Fluorescence was recorded by two-photon laser scanning microscopy on a Femto-2D microscope (Femtonics), equipped with a tunable, Verdi-pumped Ti:Sa laser (Chameleon Ultra I, Coherent), a 60× Nikon Fluor water-immersion objective (NA 1.0; Nikon Instruments), three detection channels [green fluorescence (epi and trans), red (epi), and infrared light (trans)] and controlled by MES v4.5.613 software (Femtonics).

VP-eGFP cells were identified in the green channel at an excitation wavelength of 950 nm. VPC bodies were patched in whole-cell mode with patch pipettes filled with regular intracellular solution (see Electrophysiology). AlexaFluor 549 (50 µm; Invitrogen) and the Ca^2+^ indicator OGB-1 (100 µm; Invitrogen) were added for neurite visualization and calcium imaging. Fluorescence transients and image stacks were acquired at 800 nm laser excitation. Data were mostly collected from the medial surface of the OB.

Ca^2+^ imaging experiments were performed at room temperature (∼21°C). The patched VPCs were held in current clamp mode near their resting potential of −55 mV. Again, leaky VPCs with a holding current beyond −30 pA were dismissed. A shift in baseline fluorescence F_0_ > 15% between the first and the last measurement of each region of interest also led to a rejection of the experiment. Structures of interest were imaged in free line-scanning mode with a temporal resolution of ∼1 ms. At a given dendritic location, several consecutive focal line scans during somatically evoked single action potentials (APs; by an injected current step of 1000 pA for 1 ms) or AP trains (20 stimuli at 50 Hz) were recorded (duration 1.5 s), averaged and smoothed. Dendritic Ca^2+^ transients were analyzed in terms of ΔF/F relative to the resting fluorescence F_0_ (Egger et al., 2003). For extracting the distance of the Ca^2+^ measurements from the soma and the tuft origin and performing correlation analysis MES 4.5 (Femtonics) and SigmaPlot 13.0 (Systat Software) were used, respectively.

After sufficient filling of the dendritic tree (for at least 15 min), stacks of scans of the entire cell were recorded at 1 µm *z*-resolution. Each scan included three images, recorded in the red (AlexaFluor 594) and green (OGB-1) fluorescent channel and at the same time in the trans-infrared channel of the microscope, to gather information on both the dendritic tree and glomerular structure. The *x*–*y* resolution was 900 × 900 pixels with a pixel width of 0.197 µm. All tufts fit within one scanning window and were fully sampled. In some instances, we noted on reconstruction that cells had been incompletely scanned, mostly because the stack’s *z*-coordinate was not set deeply enough. These neurons were not used for morphological analyses.

### Histology

To chemically label dendritic and axonal processes of VPCs for later investigation by light microscopy and to verify the lack of lateral dendrites of eTCs, in some of the electrophysiological experiments biocytin (5 mg/ml) was added to the intracellular solution. Slices were postfixed overnight at 4°C in 4% paraformaldehyde. Afterward, slices were stored up to 2 weeks at 4°C in 0.1 m PB (80 mm Na_2_HPO_4_, 20 mm NaH_2_PO_4_, pH 7.4) until further processing.

Staining was performed according to the protocol proposed by Marx et al. (2012). Briefly, slices were washed in PB (6–8 × 10 min). Then endogenous peroxidase activity was quenched via incubating slices for 45 min in 3% H_2_O_2_ (in PB). Again, the slices were washed for approximately three times in PB until no more bubbles were visible. Slices were incubated in ABC Solution (VECTASTAIN Elite ABC-Peroxidase Kit, Vector Laboratories) in the dark for 60 min at RT and then overnight at 4°C, followed by several washing steps in the dark (3 × 10 min in PB, then 3× in 0.05 m TrisHCl, pH 7.6). Before starting the peroxidase reaction slices were incubated in DAB solution [3,3′-diaminobenzidine (0.02%), CoCl_2_ (0.002%), NH_4_NiSO_4_ (0.004%), in TrisHCl pH 7.4]. To start the peroxidase reaction we added 3% H_2_O_2_ to the DAB solution (∼60 s, until staining was sufficiently strong), the reaction was then stopped in 0.1 m PB, and the slices were washed finally in 0.1 m PB (6–8 × 10 min). Subsequently the slices were mounted on objective slides using Moviol as mounting medium (6 *g* glycerol, 2.4 *g* Moviol 4-88, 12 ml 200 mm TrisHCl, pH 8.5, 6 ml H_2_O).

Additionally, *in vitro* slices containing biocytin-filled eGFP VPCs were postfixed as described and prepared for fluorescent double-labeling. Briefly, free-floating slices were washed in PBST (0.3% Triton-X; 3 × 10 min) and incubated for 60 min in PBST containing 5% NGS (Normal Goat Serum S-1000; Vector Laboratories). Sections were incubated with the diluted primary VP-neurophysin antibody (1:100; PS41, kindly provided by Dr. Harold Gainer, NIH, Bethesda, MD; Ben-Barak et al., 1985; Bader et al., 2012) for 48 h at 4°C. After three rinses for 10 min in PBST, the bound primary antibodies were visualized using goat anti-mouse antibodies conjugated to CF633 (1:1000; Biotium) diluted in PBST/5% NGS for 2 h at room temperature. Following washing in PBST (3 × 10 min) slices were finally incubated in streptavidin conjugated to CF488A (1:400; Biotium) for 1 h at room temperature followed by incubation overnight at 4°C and 1 h at room temperature. Following final washing steps (PBST; 3 × 10 min) the slices were mounted in objective slides using DAPI Fluoromount-G (SouthernBiotech).

Both biocytin-DAB stains of dendritic and axonal structures of VPCs as well as fluorescent double-labeling were imaged on an inverted confocal laser scanning microscope (Leica TCS SP8, Leica Microsystems). Digital images were processed (Merging and *Z*-projections) using the Leica Application Suite X and Fiji (Schindelin et al., 2012). The detailed morphology of the lateral dendrites and axonal structures of VPCs was reconstructed and analyzed with the Fiji plugin Simple Neurite Tracer (Longair et al., 2011) from the *z*-stack. Although in light microscopy thin spineless dendritic branches of juxtaglomerular cells can be mistaken for axons and vice versa, especially within the glomerular layer and superficial EPL (Kiyokage et al., 2010), classification of dendrites and axons was achieved based on the observation that all deeper projections into the mitral cell layer (MCL) clearly resemble axons in their appearance and all diverge from one single process extending directly from the soma or a thick dendritic neurite near the soma. From this analysis the number of branch points and the average branch length of dendritic and axonal arborizations were extracted. Further, the projection area of the dendritic/axonal structures in the GL/EPL as well as in the MCL/granule cell layer (GCL) was determined by measuring the area of the smallest obtuse polygon that inscribes these structures in a *z*-projection of the reconstructed VPC. The reconstructed VPCs were classified as type I or type II depending on how many times their projections cross the MCL from the EPL (type I, multiple times; type II, one time). Collaterals crossing back from the GCL to the EPL were not counted. Cells with axons that did not cross the MCL at all were dismissed as these axons clearly were truncated because of slicing.

### Reconstruction and analysis of apical tufts and glomerular shape

Reconstruction and analysis of dendritic tuft-like structures and glomeruli was performed as previously described in detail by Bywalez et al. (2016). Briefly, the detailed morphology of the apical tuft of VPCs and MCs was reconstructed with the Fiji plugin Simple Neurite Tracer (Longair et al., 2011) from the fluorescence *z*-stack scans of the Ca^2+^ imaging experiment. The glomerular contours were reconstructed from the trans-infrared image stacks with the ImageJ plugin TrakEM2 (Cardona et al., 2012). The glomerular arborization patterns of reconstructed dendritic tufts were analyzed by custom-written software based on IGOR Pro 5.0 (Wavemetrics; Bywalez et al., 2016). The aligned representations were used to determine the density and fraction of branch points within shells of the glomerulus. For analyzing the relation between the apical tuft of a VPCs and its surrounding glomerulus, five shell volumes were calculated based on the real glomerular shape via shrinking of the reconstructed glomerular surface by steps of one-fifth of the radius from the center of mass of the glomerulus. The density of branch points within a shell was determined by dividing the number of branch points by the volume of the glomerular shell in which they are located. The fraction of branch points was determined by normalization of the branch point number in a certain shell to the total number of branch points in the whole tuft. To better illustrate these data, we put them into the context of other well known glomerular dendritic structures by including a data set from rat MC apical dendritic tufts and their surrounding glomeruli. MCs had been filled with AlexaFluor 594 (50 µm; wild-type rats, P12–P16).

### Statistical analysis

Statistics was performed using SPSS 22.0 (IBM) and G*Power 3.1.9.2 (Franz Faul, University of Kiel, Kiel, Germany). Significance was accepted at *p* < 0.05. For details, see Table 1.

**Table 1.** Statistical table

	Data structure	Type of test	Power (Calculated for α = 0.05)
a	Normal distribution	T-test for independent variables (cell type [independent])	1.00 (sag)1.00 (rebound)
b	Normal distribution	ANOVA (cell type [independent]) followed by a *post-hoc* comparison using Bonferroni correction	1.00 (τ_m_)1.00 (R_i_)0.993 (threshold)0.999 (CV of ISI)1.00 (spike ratio)1.00 (AHP ratio)0.993 (AP FWHM)1.00 (AP AHP)
c	Normal distribution	T-test for independent variables (cell type [independent])	0.515
d	Normal distribution	2 × (2) mixed model ANOVA (cell type [between subject] × neurite type [within-subject]) followed by a *post-hoc* comparison using Bonferroni correction.	0.795 (cell type)
e	Normal distribution	2 × (3) mixed model ANOVA (cell type [between subject] × layer [within-subject]) followed by a *post-hoc* comparison using Bonferroni correction.	0.795 (cell type)
f	Normal distribution	2 × mixed model ANOVA (cell type [between subject] × neurite type [within-subject]) followed by a *post-hoc* comparison using Bonferroni correction.	1.00 (neurites)0.611 (cell type)0.526 (interaction)
g	Normal distribution	2 × (3) mixed model ANOVA (cell type [between subject] × layer [within-subject]) followed by a *post-hoc* comparison using Bonferroni correction.	0.884 (layer)
h	Normal distribution	T-test for independent variables (cell type [independent])	0.994 (τ_m_)
i	Normal distribution	2 × (5) mixed model ANOVA (cell type [between subject] × shell segment [within-subject]) followed by a *post-hoc* comparison using Bonferroni correction.	0.820 (cell type)
j	Normal distribution	2 × (5) mixed model ANOVA (cell type [between subject] × shell segment [within-subject]) followed by a *post-hoc* comparison using Bonferroni correction.	0.058 (cell type)
k	Normal distribution	Linear Regression [ΔF/F_0_ [dependent], distance [independent])	0.927 (dendrite)0.121 (tuft)
l	Normal distribution	ANCOVA (cell type [response variable], distance from soma [covariate])	1.00 (AP)1.00 (50 Hz)
m	Normal distribution	T-test for dependent variables (treatment [dependent])	1.00
n	Normal distribution	2 × (8) mixed model ANOVA (treatment [between subject] × time [within-subject]) followed by a *post-hoc* comparison using Bonferroni correction	0.935 (treatment)0.610 (time)0.945 (interaction)
o	Normal Distribution	2 × (8) mixed model ANOVA (treatment [between subject] ×time [within-subject]) followed by a *post-hoc* comparison using Bonferroni correction	0.999 (treatment)0.971 (time)1.00 (interaction)
p	Normal distribution	Repeated measures ANOVA (treatment [dependent])	0.996 (treatment)

## Results

### Electrophysiological properties of VPCs

**Figure 1.**
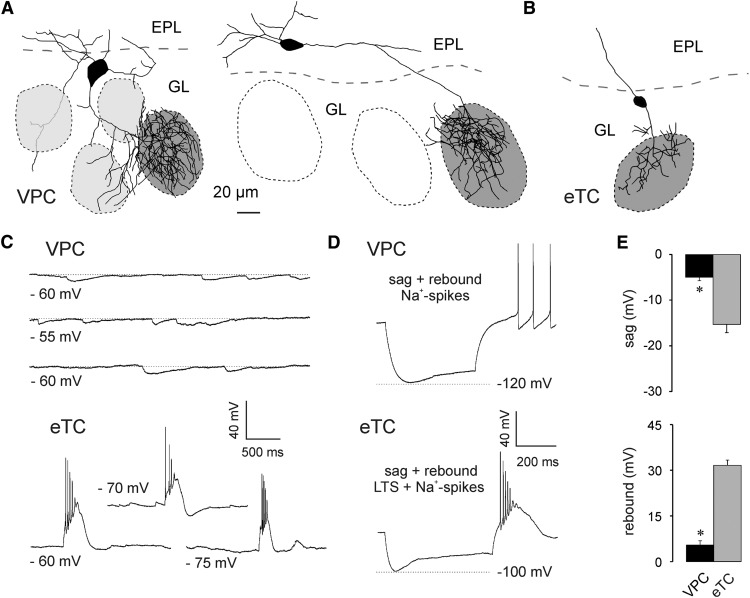
***A***, Two representative VPC reconstructions. The dark gray shading indicates the glomerulus innervated by the dendritic tuft of the respective VPC. VPCs bear several lateral dendrites that either run below the GL or lie above or underneath other glomeruli (light gray shading). ***B***, Representative reconstruction of an eTC. ***C***, Representative spontaneaous IPSPs and bursts of 3 different VPCs and eTCs, respectively. ***D***, Representative responses to somatically applied current steps (−90 to −100 pA; 800 ms) to VPCs and eTCs. ***E***, Cummulative comparison of the sag (top) and the rebound depolarization/LTS (bottom) of VPCs (*N* = 23) and eTCs (*N* = 17). * *p* < 0.001 versus eTC. *T*-test for independent variables. Data are mean ± SEM.

To characterize the electrophysiological properties of VPCs and to investigate potential differences from other large glutamatergic bulbar neurons, we systematically performed current-clamp *in vitro* recordings from eGFP-labeled VPCs and other tufted glutamatergic cells in the OB, i.e., MC, mTCs, and eTCs, that were identified based on the location and size of their somata. The identity of eTCs was further verified by biocytin-DAB staining to confirm the lack of lateral dendrites (Fig. 1*A*,*B*).

Whole-cell current-clamp recordings at resting *V*_m_ sometimes revealed spontaneous IPSP activity in VPCs (Fig. 1*A*,*C*; 16 of 37 cells from 26 rats). Only 1 of the 37 VPCs showed small spontaneous EPSPs, whereas bursting activity was never observed. In contrast, eTC recordings always contained spontaneous EPSPs and often also the characteristic spontaneous AP bursts (10 of 18 cells from 12 rats; Hayar et al., 2004a) or low threshold spikes (LTSs; 4 of 18 cells; Fig. 1*B*,*C*).

In both VPCs (*N* = 23 from 23 rats) and eTCs (*N* = 17 from 12 rats) application of strongly hyperpolarizing current steps (−90 to −100 pA) resulted in the expression of a sag (Fig. 1*D*,*E*), followed by a small rebound depolarization in VPCs or bursting (LTS + spikes) in eTCs. In 9 of 23 VPCs the rebound depolarization resulted in rebound spiking (Fig. 1*D*). Both, sag amplitude (*t*_(38)_ = 6.35, *p* < 0.001) and rebound depolarization (*t*_(38)_ = −12.2, *p* < 0.001)_a_ of VPCs (*N* = 23) were significantly smaller than the sag amplitude and the LTS component of eTCs (*N* = 17).

**Figure 2.**
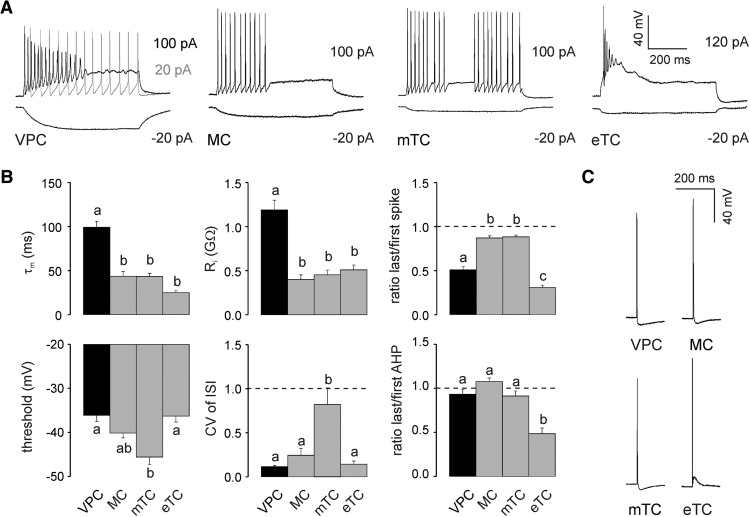
***A***, Representative responses to somatically applied current steps (600–800 ms) to VPCs, MCs, mTCs, and eTCs at their corresponding resting potential (−55 , −70 , −70 , and −60 mV, respectively). ***B***, Cumulative comparison of the membrane time constant (τ_m_, *N* = 24/25/18/18), input resistance (R_i_, *N* = 24/25/18/18), firing threshold (*N* = 24/23/18/18), CV of ISI (*N* = 22/13/13/7), last/first spike amplitude ratio (*N* = 24/13/13/11), and last/first afterhyperpolarization (AHP; *N* = 24/13/13/11) amplitude ratio measured from corresponding current step responses (see ***A***). ***C***, Representative APs evoked by somatic current injection (1000 pA, 1 ms). Arabic letters above columns illustrate whether means are statistically different (e.g., a versus b vs c) or not (e.g., a versus a vs ab). One-way ANOVA followed by *post hoc* comparison using Bonferroni correction. Data are means ± SEM.

Application of depolarizing current steps (80–120 pA) to VPCs in whole-cell patch-clamp recordings resulted in regular, non-bursting firing patterns with a slight adaption in spike amplitude (*N* = 24 from 20 rats) that were similar to the regular, non-bursting MC firing patterns (*N* = 25 from 23 rats), but clearly distinguishable from the irregular patterns of mTCs and bursting eTCs (*N* = 18 + 18 from 10 + 12 rats; Fig. 2*A*). The regularity of the VPC firing pattern showed in its CV of ISI, because the VPCs’ CV of ISI was comparable to that of MCs but significantly lower than that of irregularly firing mTCs (*F*_(3,51)_ = 11.4, *p* < 0.001; *N* = 55; Fig. 2*B*)_b_. Note that already small depolarizing current injections (20 pA) were able to induce continuous firing in VPCs (*N* = 31 from 30 rats; Fig. 2*A*), in contrast to the adaption observed at higher current injections. The lack of bursting in VPCs was reflected in their significantly higher last/first spike amplitude ratio and AHP amplitude ratio compared with those of bursting eTCs (spike ratio: *F*_(3,57)_ = 64.7, *p* < 0.001; *N* = 61; AHP ratio: *F*_(3,57)_ = 14.4, *p* < 0.001; *N* = 61; Fig. 2*B*)_b_.

Current pulse application (1000 pA, 1 ms) resulted in APs in VPCs that were similar in amplitude to the other cell types tested (Fig. 2*C*). However, VPC APs were significantly broader [full-width at half-maximum (FWHM): VPC, 1.7 ± 0.06 ms; MC, 1.5 ± 0.05 ms; mTC, 1.3 ± 0.5 ms, eTC, 1.5 ± 0.07 ms; *F*_(3,81)_ = 8.72, *p* < 0.001, *N* = 85]_._ VPC AHPs were similar to those of mTCs and MCs and clearly different from the afterdepolarization observed in eTCs (VPC: −6.5 ± 0.79 mV; MC: −7.0 ± 0.50 mV; mTC: −5.9 ± 0.72 mV, eTC: 7.7 ± 1.2 mV; *F*_(3,73)_ = 71.8, *p* < 0.001, *N* = 77)_b_.

Hyperpolarizing current steps (−20 to −10 pA) elicited slowly hyperpolarizing voltage responses from VPCs compared with the faster hyperpolarization in MCs and mTCs or the very fast hyperpolarization in eTCs (Fig. 2*A*). Accordingly, the membrane time constant (τ_m_) of VPCs was >2 times higher than that of MCs, mTCs, and eTCs (*F*_(3,81)_ = 37.9, *p* < 0.001; *N* = 85; Fig. 2*B*)_b_. Besides the high τ_m_, the input resistance (R_i_) in VPCs was also >2 times higher than the R_i_ of the analyzed MCs, mTCs and eTCs (*F*_(3,81)_ = 27.1, *p* < 0.001; *N* = 85; Fig. 2*B*)_b_. Regarding the spiking threshold VPCs did not differ from MCs and eTCs. However, their spiking threshold was significantly higher than that of mTCs (*F*_(3, 74)_ = 8.77, *p* < 0.001; *N* = 78; Fig. 2*B*)_b_. In summary, although VPCs showed a slow τ_m_ they were still as excitable as the other TCs because their high R_i_ compensates for the sluggish polarization.

In conclusion, the electrophysiological properties of VPCs, especially the lack of bursting, suggest an overlap of VPCs with the population of juxtaglomerular tufted cells (TC) with lateral dendrites described by Antal et al. (2006).

### Subcellular VP expression in VPCs

**Figure 3.**
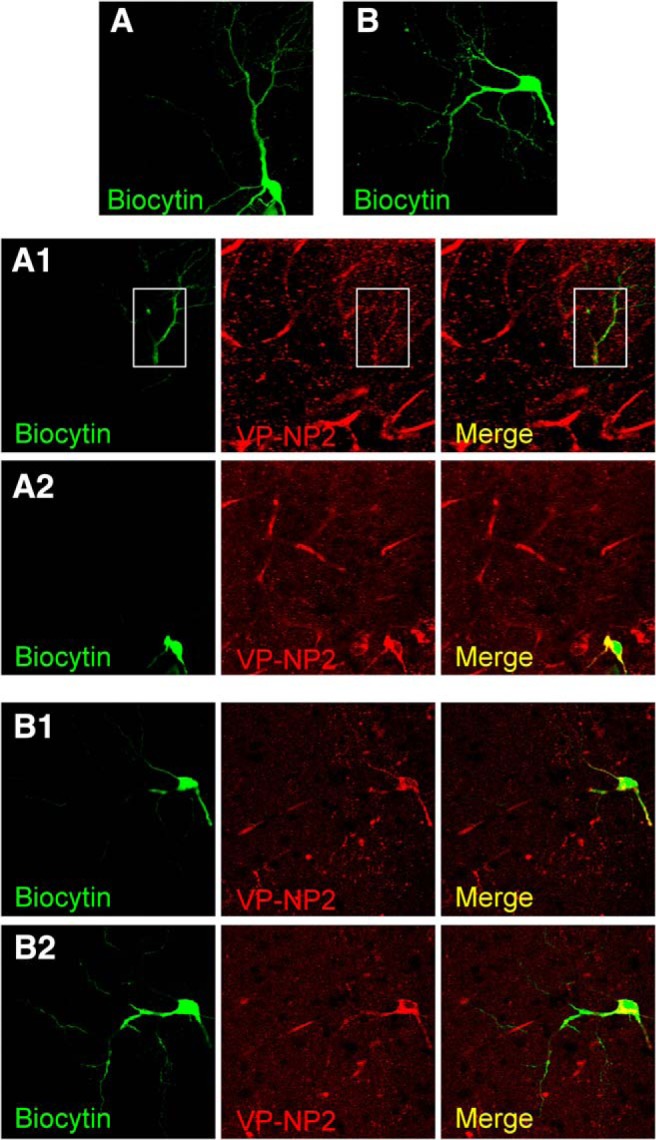
***A***, ***B***, Average *z*-projections of eGFP-VPCs labeled with biocytin (visualized with streptavidin-conjugated CF488A) and corresponding staining of VP-Neurophysin 2 (VP-NP2, CF633).

The local presence of VP protein is a prerequisite for local VP release. In hypothalamic VPCs VP is known to be expressed within and released from their soma, dendrites, and axon (Pow and Morris, 1989). To investigate the actual expression of VP in the different substructures of OB VPCs, we double-stained streptavidin-fluorophore-enhanced biocytin-filled eGFP-labeled VPCs for VP/neurophysin. Unfortunately, the fluorescent labeling for VP/neurophysin could not be visualized in all thin axonal structures or the thin ramifications of the apical tuft (Fig. 3*A*,*B*). However, the double-staining clearly demonstrated that VP/neurophysin is expressed in the lateral dendrites and the origins of axonal structures (Fig. 3*A2*,*B1*,*B2*) as well as in the proximal thick branches of the apical tuft (Fig. 3*A1*) and the soma. Thus, all compartments of VPCs are potential release sites. In the following sections, we examine the different morphological compartments more closely.

### Morphology of lateral dendrites and axons

VP-binding VP and oxytocin receptors have been localized in the GL, EPL, MCL, and GCL of the OB (Ostrowski et al., 1994; Vaccari et al., 1998; Tobin et al., 2010). However, it is unknown so far whether neurites of VPCs are sufficiently proximal to all these receptor locations to release VP onto them. Because fluorescent dyes often cannot properly visualize thin neuronal processes, in particular axons (Bywalez et al., 2016), we filled VPCs with biocytin and in a first step reconstructed the lateral dendrites and axons before focusing on the prominent apical tuft.

We found that in VPCs an average of 3.7 ± 0.5 (*N* = 19 from 18 rats) lateral dendritic branches originated from their somata. All these cells had at least one (*N* = 1) or more lateral dendrites (*N* = 18).

**Figure 4.**
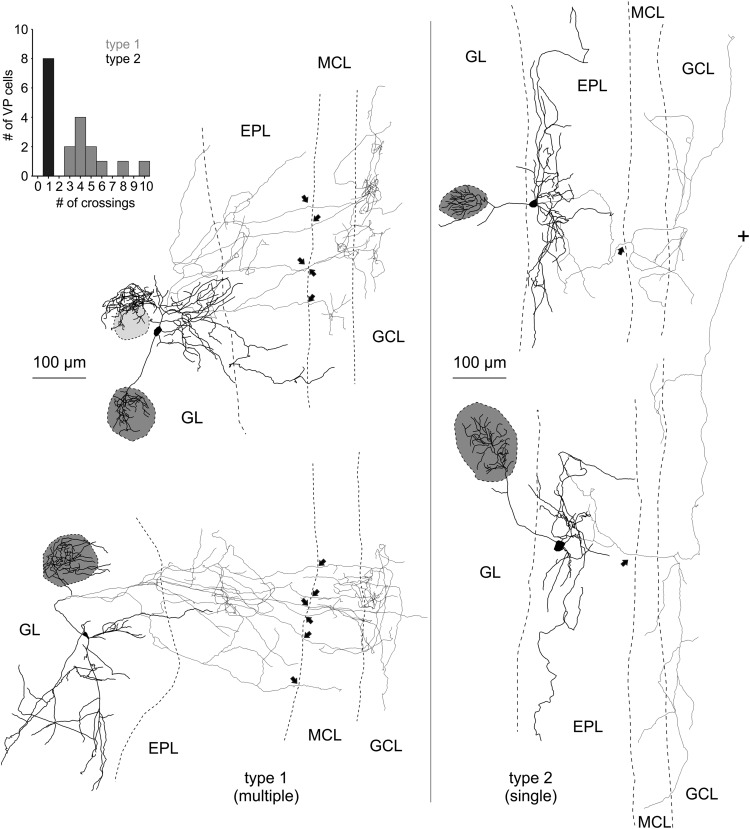
Reconstructions of eGFP-VPCs labeled with biocytin. Two representative examples of type 1 (multiple innervation of MCL; left) and type 2 cells (single top-down innervation of MCL; right). +, Truncation of axonal projection; arrows, site of axonal MCL crossing from EPL into GCL. The dark gray shading indicates the glomerulus innervated by the dendritic tuft of the respective VPC. Light gray shading indicates that dendrites lie above or below respective glomerulus. The VPC in the top left displays a conspicuous dendritic ramification that does not enter the adjacent glomerulus. Similar structures were found in 4 of 35 reconstructed cells. Inset, Number of crossings versus number of cells.

The detailed dendritic and axonal reconstructions indicated the existence of two subtypes of VPCs depending on whether their axon innervates the MCL via multiple projections (type 1) or via one main collateral (type 2; Fig. 4, left), because the number of crossings into the MCL was bimodally distributed (*N* = 19; Fig. 4, inset). There was no significant difference in soma size or in the distribution of the somata across the GL and EPL between the two types (Table 2). Although type 1 had significantly less lateral dendrites than type 2 (*t*_(17)_ = −2.41, *p* = 0.028; Table 2)_c_, the projection areas of the dendritic and axonal structures did not differ between the two types (Table 2). Also, there were no differences between the two projection types in the number of dendritic branch points or average dendritic branch length (Table 2), but they were significantly different concerning the distribution of their axons below the GL. Type 1 showed a significantly higher number of axonal branch points (*post hoc*: *p* = 0.003; cell type effect: *F*_(1,17)_ = 8.73; *p* = 0.009; *N* = 11/8 from 18 rats; Table 2)_d_. When comparing the axonal branch points of the two types with regard to their distribution within the layers of the OB, type 1 had a significantly higher number of axon branch points in the GL and EPL than type 2 (*post hoc*: *p* < 0.001; cell type effect: *F*_(1,17)_ = 8.73; *p* = 0.009; *N* = 11/8 from 18 rats; Table 2)_e_. In contrast, the average axonal branch length of type 1 was significantly lower than that of type 2 (*post hoc*: *p* = 0.022, cell type effect: *F*_(1,17)_ = 5.66; *p* = 0.029; *N* = 11/8 from 18 rats; Table 2)_f_.

**Table 2.** Electrophysiological and morphological properties of type 1 and type 2 VPCs

	Type 1 (*N* = 11)			Type 2 (*N* = 8)		
Pre-selection parameterCrossings MCL	5 ± 0.7			1		
Neurites	Dendrites	Axon		Dendrites	Axon	
Layers	GL + EPL	GL + EPL	MCL + GCL	GL + EPL	GL + EPL	MCL + GCL
Branchpoints^d,e^	32 ± 5.5	23 ± 2.4*	17 ± 6.0	28 ± 4.4	4.1 ± 1.0^#^	6.6 ± 2.7^#^
	32 ± 5.5	40 ± 6.8*		28 ± 4.4	11 ± 3.1	
Branch length (µm)^f,g^	40 ± 3.4	63 ± 5.4^#^	68 ± 8.8	46 ± 5.3	81 ± 11^#^	117 ± 29^#^
	40 ± 3.4	62 ± 5.9*^#^		46 ± 5.3	93 ± 12^#^	
Area neurites (mm^2^)	0.05 ± 0.01	0.5 ± 0.3		0.05 ± 0.01	0.11 ± 0.04	
Area soma (µm^2^)	146 ± 25			182 ± 26		
Location soma	8 × GL vs. 3 × EPL			2 × GL vs. 6 × EPL		
Lateral dendrites^c^	3 ± 0.4*			5 ± 0.3		
τ_(m)_, (ms)^h^	65 ± 6.9*			96 ± 6.3		
R^i^ (MΩ)	530 ± 78.2			927 ± 204		
Threshold (mV)	−50 ± 1.7			−48 ± 1.2		
AP amplitude (mV)	76 ± 2.1			67 ± 5.6		
Last/first spike ratio	0.73 ± 0.04			0.66 ± 0.08		
Last/first AHP ratio	1.1 ± 0.04			0.88 ± 0.11		

Mixed-model ANOVA followed by a *post hoc* comparison *d*, *e* , *f*, *g* using Bonferroni correction or *t*-test for independent samples *c*, *h*; * *p* < 0.05 versus type 2; # *p* < 0.05 versus dendrites. Data are mean ± SEM.

Interestingly, the two projection types also differed with respect to an electrophysiological parameter, their membrane time constant (τ_m_): type 1 cells had a significantly faster τ_m_ than type 2 cells (*t*_(16)_ = −3.09, *p* = 0.007; *N* = 11/7 from 18 rats; Table 2)_h_. The R_i_, spiking threshold, spike amplitude, spike ratio, and AHP ratio were not different between the two morphological groups (Table 2).

A comprehensive analysis of the axonal morphology including also non-reconstructed VPCs revealed a much higher overall prevalence of type 1 (*N* = 63) compared with type 2 (*N* = 10).

In summary, type 1 more densely (more branch points) innervates the superficial layers with its axon and features multiple but short local projections (shorter branch length) to the deeper layers, i.e., MCL and superficial GCL. type 1 axonal projections are thus more prominent directly medial to the home glomerulus, probably interacting with the respective glomerular column (Willhite et al., 2006). Conversely, type 2 has a more sparse overall axonal innervation (less branch points) in total but has wider-ranging projections (longer branch length), especially below the MCL reaching either deeper into the GCL or alongside the internal plexiform layer to more distant targets (Fig.4).

Because the biocytin-DAB staining that was used for the axon visualization relies on postfixation and extensive *post hoc* histochemical treatment the reconstructions suffer from tissue shrinkage, especially in the *z*-direction of the slice (Egger et al., 2008). This effect complicates the reconstruction of the very dense structure of the apical dendrite/tuft of VPCs. Thus, we reconstructed the tufts of eGFP-labeled VPCs filled with fluorescent dye from unfixed slices along with their “home glomeruli” as described previously for juxtaglomerular neuron types (Bywalez et al., 2016).

### Morphology of the apical tuft

**Figure 5.**
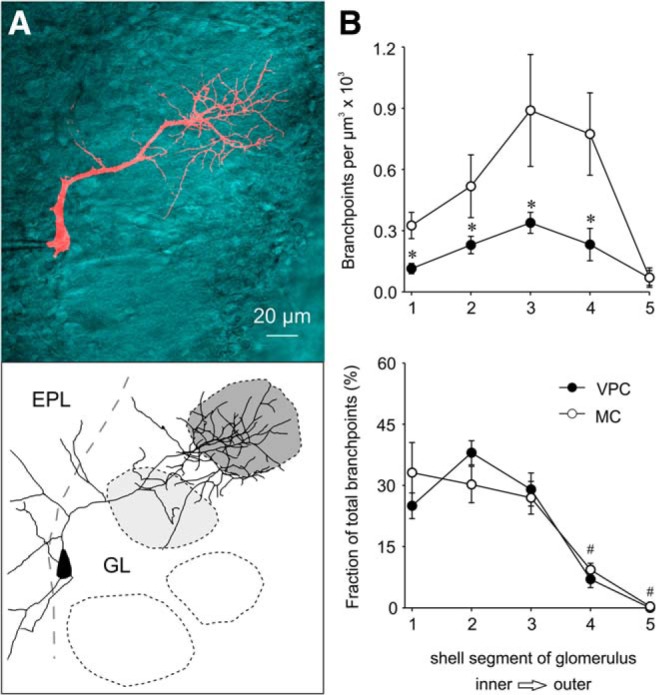
***A***, Maximal *z*-projection of a VPC filled with AlexaFluor 594 and overlaid with the single *z*-plane of the trans-infrared channel that showed the maximal extent of the innervated glomerulus. Bottom, Reconstruction of cell above. The dark gray shading indicates the glomerulus innervated by the dendritic tuft of the respective VPC. The other glomeruli have no contact with the VPC (white) or lie above or underneath the dendrites of the VPC (light gray shading). Imaging was performed in acute slices (300 µm) using two-photon laser scanning microscopy. ***B***, Density of branch points and fraction of total branch points within shell segments of the respective glomerulus in VPCs (*n* = 13) and MCs (*n* = 8). Statistical comparisons indicate a lower branch point density but similar branch point distribution in VPCs compared with MCs. * *p* < 0.05 versus respective MC; # *p* < 0.05 versus segment 1 + 2; mixed-model ANOVA followed by *post hoc* test with Bonferroni correction. MC, mitral cell; VPC, vasopressin cell.

Using two-photon microscopy *z*-projections of fluorophore-filled VPCs, we were able to reconstruct and characterize the branching patterns of the glomerular innervation by the apical dendrite/tuft of VPCs and compared them to MCs. In contrast to the rather straight apical dendrites of MCs and mTCs, VPCs’ apical dendrites (length 109.1 ± 13.3 µm, *N* = 13 from 10 rats) often take a tortuous route around neighboring glomeruli to innervate one single glomerulus with a tuft-like structure (Figs. 1*A*, 5*A*). All VPC tufts showed a uniform, widespread innervation of their “home glomerulus” (Figs. 1*A*, 5*A*). The neighboring glomeruli are not innervated, because also lateral VPC dendrites were not found to enter them (Figs. 1*A*, 5*A*). To quantify the glomerular innervation pattern of the apical dendritic tuft, we measured the density of branch points and fraction of total branch points within shell segments of the respective glomerulus in VPCs (*N* = 13 from 10 rats) and MCs (*N* = 8 from 8 rats; see Materials and Methods; Bywalez et al., 2016). VPCs had a significantly lower branch point density (*F*_(1,19)_ = 9.20, *p* = 0.07)_i_ but a similar branch point distribution across their glomerular shells (*F*_(1,19)_ = 0.080, *p* = 0.780)_j_ compared with MCs (Fig. 5*B*). Thus, similar to MCs, VPC tufts would be in a position to both receive inputs and provide output throughout the whole glomerulus; in contrast to classical eTCs that fan out in only part of the glomerulus (Fig. 1*B*; Pinching and Powell, 1971)

This dense innervation of its home glomerulus by the apical tuft, along with the subcellular VP expression (Fig. 3) and the presence of VP-receptive VP and oxytocin receptors throughout the glomerular layer (Vaccari et al., 1998; Manning et al., 2008; Tobin et al., 2010), implies a functional role of the VPC tuft as a potential site of release for VP.

### Tuft excitability as established by backpropagating APs

**Figure 6.**
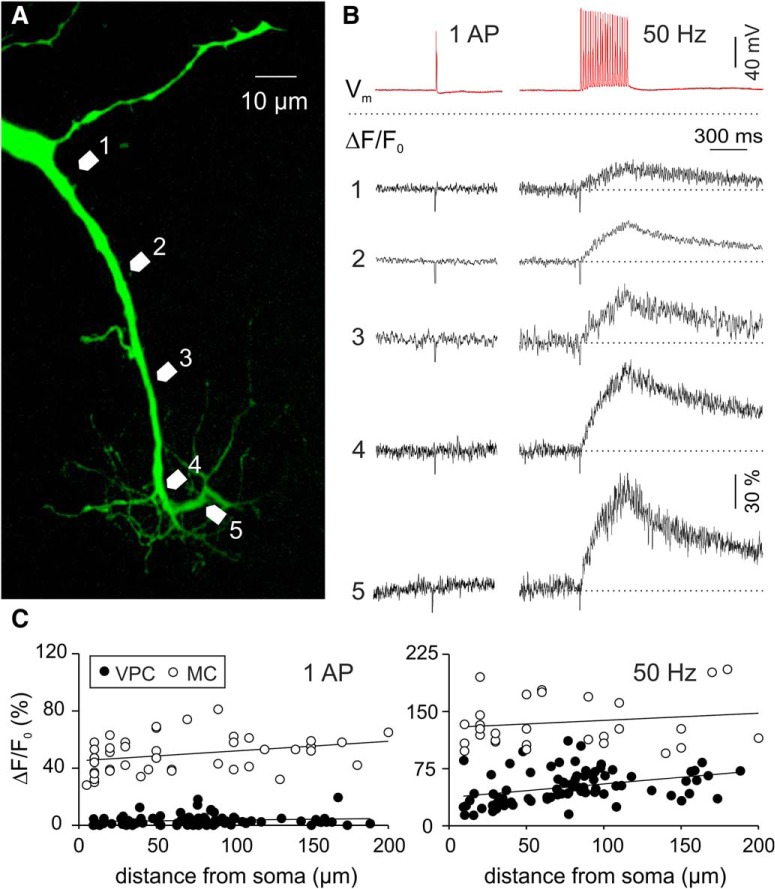
***A***, Two-photon scan of a representative VPC filled with the Ca^2+^-sensitive dye OGB-1. Numbered arrows in the scan correspond to the locations of the numbered averaged (*n* = 4) ΔF/F transients shown in ***B***, in response to a single somatically-evoked AP (1000 pA, 1 ms) or a 50 Hz train (20 APs, 50 Hz, 400 ms). ***C***, ΔF/F of apical dendrites and tufts versus distance from soma in VPCs (*N* = 11) and MCs (*N* = 13). MC, mitral cell; VPC, vasopressin cell.

Neurons in the OB that are capable of dendritic release usually feature strong AP backpropagation from the soma, which is accompanied by substantial dendritic Ca^2+^ entry. Such Ca^2+^ signals were observed in apical dendrites of GCs and lateral dendrites, apical dendrites and tufts of MCs (Xiong and Chen, 2002; Debarbieux et al., 2003; Egger et al., 2003). Therefore we hypothesized that VPCs’ tufts would be similarly excitable. We imaged Ca^2+^ signals in response to backpropagating somatically evoked single APs (sAPs) and trains (20 APs at 50 Hz) within the apical dendrite and tuft. Surprisingly, we consistently observed very small or no dendritic Ca^2+^ transients in response to sAPs (tuft ΔF/F amplitude: 3.9 ± 0.8%, *n* = 38 measurements in *N* = 11 cells from 9 rats; soma/apical dendrite below tuft ΔF/F: 2.7 ± 0.4%, *n* = 42/*N* = 11 from 9 rats; Fig. 6*B*). Trains caused a moderate rise in ΔF/F (tuft: mean amplitude 56 ± 3.0%, *n* = 38/*N* = 11; soma/dendrite: 46 ± 3.6%; Fig. 6*B*), which demonstrates that voltage-gated Ca^2+^ channels are indeed present in the tuft. These ΔF/F responses to trains significantly increased along the apical dendrite (*R* = 0.475, *R^2^* = 0.225, *p* = 0.001, *n* = 42/*N* = 11)_k_, but only until the main branch point of the glomerular tuft (*R* = −0.128, *R^2^* = 0.016, *p* = 0.445, *n* = 38/*N* = 11)_k_.

To control for the small size of sAP-mediated Ca^2+^ signals in VPCs (*N* = 11 from 9 rats) we compared these data to a corresponding data set of MCs (*N* = 13 from 10 rats) recorded with the same technique (Egger and Stroh, 2009). In these cells, single APs as well as prolonged trains produced substantial, significantly higher Ca^2+^ signals than in VPCs (sAP: F_(1,125)_ = 1035; *p* < 0.001; *n* = 128/*N* = 24; 50 Hz: F_(1,107)_ = 268; *p* < 0.001; *n* = 110/*N* = 24; Fig. 6*C*)_l_.

In conclusion, in terms of Ca^2+^ entry VPC tufts appear much less responsive to propagating APs than MC tufts. Therefore sAPs are highly unlikely to admit an amount of Ca^2+^ sufficient for VP release from the dendrite. However, this observation does not exclude the possibility that synaptic inputs, e.g., from the ON can provide local synaptic excitation and thus substantial local Ca^2+^ entry (as known for MC tufts; Yuan and Knöpfel, 2006) that could trigger VP release from VPCs in a local reciprocal manner.

### ON-mediated inputs to VPCs

Tufted glutamatergic MCs, mTCs, and eTCs receive monosynaptic and/or disynaptic excitation from the ON onto their apical dendritic tufts (Heyward et al., 2001; Hayar et al., 2004b; Burton and Urban, 2014). Therefore, we expected that ON activation would also excite VPCs. We performed whole cell recordings from VPCs and electrically stimulated the ON axons anterior to the glomeruli above the soma of the recorded VPC. Surprisingly, single ON stimulation did not result in direct excitation, but induced IPSPs (*N* = 97 VPCs from 77 rats; Fig. 6*B*). The observed IPSPs had a mean amplitude of −10.7 ± 0.6 mV (*N* = 11 from 10 rats). Their mean latency of >10 ms after ON stimulation (12.6 ± 0.8 ms) indicates a polysynaptic pathway of inhibition. ON-evoked VPC IPSPs had slow kinetics (rise time: 35 ± 3 ms, decay in terms of half duration: 254 ± 30 ms) compared with the kinetics of spontaneous IPSPs in MCs recorded under similar conditions (risetime: 12 ± 7 ms, half duration: 40 ± 15 ms; Egger and Stroh, 2009).

**Figure 7.**
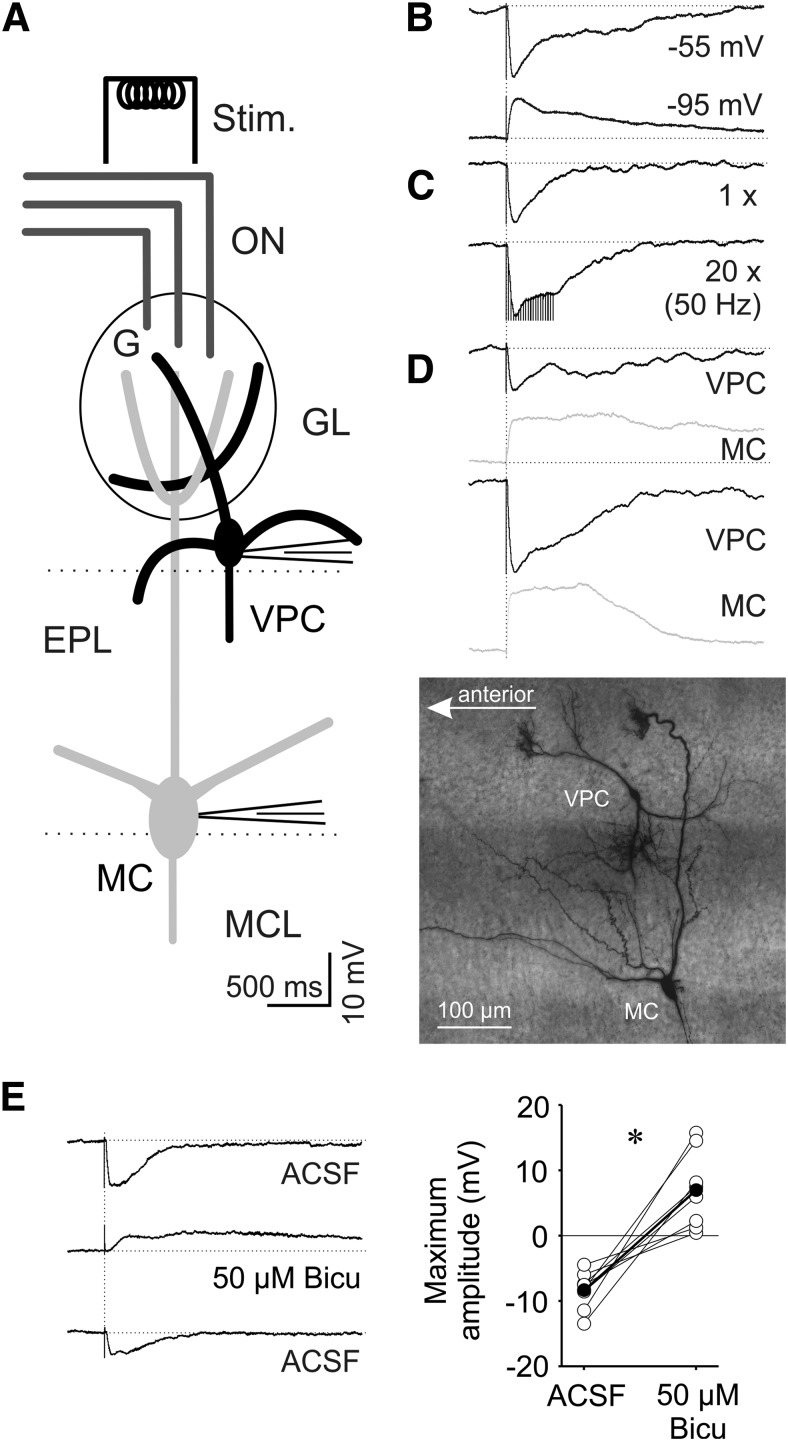
***A***, Schematic drawing of experimental setup. Whole-cell patch-clamp recordings in 300 µm *in vitro* slices of responses to electrical ON stimulation (400 µA, 100 µs, 30 s intervals). ***B***, Representative averaged (10 traces) ON-evoked IPSPs recorded from a VPC at resting potential of −55 mV (total *N* = 97) and hyperpolarized to −95 mV (total *N* = 7). ***C***, Representative averaged (10 traces) ON-evoked IPSPs stimulated one time and 20 times at 50 Hz recorded from a VPC at resting potential of −55 mV (*N* = 3). ***D***, Representative averaged PSPs from two pairs of VPCs and MCs sequentially stimulated, but at the same location within the same slice (image: maximal *z*-projection of a representative pair of a VPC and MC from this experiment visualized by subsequent biocytin-DAB staining). ***E***, Bath application of 50 µM bicuculline (competetive GABA_A_ receptor antagonist). Left, Representative averaged (10 traces) ON-evoked PSPs recorded from a VPC. Right, Cumulative presentation of bicuculline effect on PSP amplitudes (*N* = 8). Empty dots represent single measurements, whereas filled dots represent means. *p < 0.05 versus ACSF. *T*-test for dependent variables. Amplitudes of stimulus artifacts were truncated. Bicu, bicuculline; G, glomerulus.

Stronger ON stimulation with trains of current pulses (20× at 50 Hz), did also not result in an excitatory postsynaptic response (Fig. 7*C*; *N* = 3 from 3 rats). For additional confirmation of the unexpected finding of predominantly inhibitory responses we recorded ON-evoked EPSPs from MCs located proximal to a VPC that responded with IPSPs to stimulation at the same site (*N* = 4 from 4 rats; Fig. 7*D*). Therefore, it is highly unlikely that the observed IPSPs are artefacts of our stimulation technique (e.g., wrong positioning or insufficient stimulation strength) or because of other systemic parameters (ACSF, intracellular solution, etc.).

To investigate if these VPC responses are indeed GABAergic, VPCs were current-clamped from −55 to −95 mV. Responses to ON stimulation then became depolarizing, arguably because of the reversal of Cl^−^ currents through GABA receptors (Fig. 7*B*; *N* = 7 from 6 rats). Next, the GABA_A_ receptor antagonist bicuculline completely blocked IPSPs recorded at −55 mV (*t*_(7)_ = −5.48, *p* < 0.001; *N* = 8 from 8 rats)_m_ and unmasked barrages of putative EPSPs (Fig. 7*E*). These barrages had an amplitude of 6.9 ± 1.8 mV and even longer onset latencies (46.1 ± 27.5 ms) indicating a polysynaptic nature also for these inputs.

A detailed analysis of all our VPC recordings with both ON stimulation and recovered morphology revealed that 69 of the 70 VPCs (from 58 rats) that responded with IPSPs still had an intact apical tuft, whereas 11 of the 15 VPCs (from 12 rats) without or a massively cut tuft did not show any ON-evoked IPSPs. Thus, candidate inhibitory inputs should be restricted to juxtaglomerular interneurons that innervate glomeruli, e.g., periglomerular cells or “short-axon” cells. As to the excitatory barrages, 15 of the 15 VPCs without apical tuft showed either small IPSPs with no late depolarization (*N* = 4 from 12 rats) or no signal at all (*N* = 11 from 12 rats) on ON stimulation, indicating that the postsynaptic origin of this excitatory signal, like the inhibitory one, is most likely located in the apical tuft. Thus, we propose that the postsynaptic origins of both IPSP and EPSP barrage are located within the home glomerulus, i.e., on the VPC tuft.

These findings imply that ON inputs alone are unlikely to excite VPCs and thus cannot invoke glomerular VP release by themselves. Nevertheless, many cells in the glomerular layer express VP-receptive VP and oxytocin receptors (Vaccari et al., 1998; Manning et al., 2008; Tobin et al., 2010), the dendritic tuft is excitable (Ca^2+^ entry) by somatic depolarization, and VP is expressed in apical and lateral dendrites of VPCs (Fig. 3; De Vries et al., 1985). Thus, although at this point we do not know the origin of physiologically relevant excitatory stimuli that could result in glomerular VP signaling, we next investigated whether VP can indeed affect glomerular synaptic processing.

### Effects of VP on GL tufted cells (eTCs and VPCs)

If VP application had an effect on synaptic glomerular signaling, such observations could provide additional indirect evidence for a role of endogenous release of VP in glomerular processing. The fact that the dendritic compartment of eTCs consists solely of an apical tuft within one glomerulus (and no lateral dendrites; Fig. 1*B*; Hayar et al., 2005) makes them utilizable as glomerular VP sensors.

**Figure 8.**
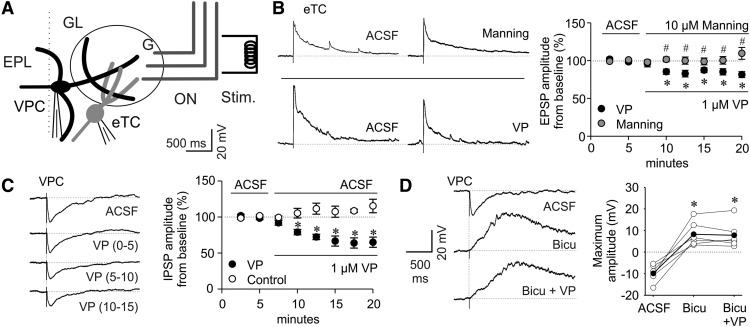
***A***, Schematic drawing of experimental setup. Whole-cell patch-clamp recordings from VPCs or eTCs of responses to electrical ON stimulation (20–400 µA, 100 µs, 30 s intervals). Bath application of 1 µM VP or 10 µM of a VP receptor antagonist (Manning compound). ***B***, Left, Representative averaged (10 traces) ON-evoked EPSPs recorded from a eTC. Right, Cumulative averaged (5 traces) presentation of VP/Manning compound effect on EPSP Amplitudes (*N* = 8/7). ***C***, Representative averaged (10 traces) ON-evoked PSPs recorded from a VPC and cumulative averaged (5 traces) presentation of 1µm VP effect on IPSP Amplitudes (*N* = 6, numbers in brackets represent time bins in minutes after VP). ***D***, Bath application of 50 µm bicuculline (competetive GABA_A_ receptor antagonist) and 1 µm VP. Left, Representative averaged (10 traces) ON-evoked IPSPs recorded from a VPC. Right, Cumulative presentation of bicuculline/VP effect on PSP amplitudes (*N* = 6). Empty dots represent single measurements, whereas filled dots represent means. Data points are mean ± SEM. *p < 0.05 versus corresponding ACSF. #*p* > 0.999 versus ACSF. *T* test for dependent variable (***D***). Mixed-model ANOVA followed by *post hoc* test with Bonferroni correction (***B***, ***C***). Amplitudes of stimulus artifacts were truncated. Bicu, Bicuculline; G, glomerulus.

To activate synaptic glomerular processing, we again used ON stimulation, and recorded from individual eTCs. As expected, eTCs responded with EPSPs (Hayar et al., 2004b), further confirming our finding of ON-evoked IPSPs in VPCs. Application of 1 µM of VP *in vitro* slightly but significantly reduced ON-evoked EPSP amplitudes to 85 ± 2.8% of baseline (interaction effect: *F*_(9,117)_ = 4.94, *p* = 0.002; *N* = 15 from 12 rats; Fig. 8*B*). This finding supports the hypothesis that endogenously released VP could exert these direct or indirect effects preferentially within the eTC’s home glomerulus and thus originate from a VPC tuft in the same glomerulus.

Further, we were interested whether ON stimulation as such is capable of causing VP release. However, application of a selective VP antagonist (10 µm Manning compound) did not modulate the amplitude of ON-evoked EPSPs in eTCs and was also significantly different from the effect of the VP application (amplitude 99 ± 3.2% of baseline; *N* = 15 from 12 rats, interaction effect: *F*_(9,117)_ = 4.94, *p* = 0.002; Fig. 8*B*)_n_. This finding implies that ON activity is unlikely to induce endogenous glomerular VP release, in line with our previous finding of predominantly inhibitory ON action on VPCs (Fig. 7). Moreover, the experiment may serve as a control against rundown of eTC EPSPs in response to extended repeated ON stimulation.

Further, to elucidate whether VPCs are capable of autocrine self-excitation like VPCs in the hypothalamus (Sabatier et al., 1997), we investigated the effects of exogenous VP on ON-evoked IPSPs in VPCs. Application of 1 µM of VP *in vitro* reduced the evoked IPSP amplitude to 69 ± 3.9% of baseline (*N* = 12 from 12 rats, interaction effect: *F*_(7,70)_ = 10.3, *p* < 0.001; Fig. 8*C*)_o_ compared with further ACSF application. This reduction of ON-evoked VPC inhibition might serve to increase the probability for VPC excitation and thus release via other pathways. Finally, during recordings of ON-evoked excitatory EPSP barrages from VPCs in the presence of the GABAergic blocker bicuculline (50 µm; Fig. 7*E*), bath application of 1 µm VP could not further increase the amplitude of the excitatory signal (*N* = 6, *F*_(2,10)_ = 32.0, *p* = 0.002; Fig. 8*D*)_p_. This indicates that VP acts on the transmission of GABAergic interneurons, but rather not on excitatory inputs to VPCs, like eTCs, mTCs, MCs, and ON, as otherwise the isolated EPSP barrages would have been also modulated by VP.

## Discussion

### VPCs as superficial tufted cells

Our detailed investigation revealed that VPCs feature several unique electrophysiological and anatomic properties that differentiate them from other glutamatergic tufted cell types in the OB. In the initial study by Tobin et al. (2010) VPCs were considered as classical eTCs, based on the observation of bursting firing patterns and spontaneous bursts that characterize classical eTCs without lateral dendrites (Hayar et al., 2004b). In our study VPCs always featured non-bursting, regular firing patterns and lateral dendrites. Notably, classical eTCs have been described to reside in the GL (Hayar et al., 2004b), whereas TCs located at the border between EPL and GL including the superficial part of the EPL, as observed here for VPCs, are often referred to as superficial tufted cells (sTCs; Hamilton et al., 2005; Nagayama et al., 2014; Tavakoli et al., 2018). Just like sTCs, VPCs bear several lateral dendrites that spread in the EPL and an apical dendrite that takes a tortuous route through the GL before entering its home glomerulus and forming a tuft. By comparison, classical eTCs feature a tuft that originates almost directly from the soma, and lack lateral dendrites. Also, the VPC apical tuft branching pattern inside the glomerulus shows a uniform, widespread innervation very similar to that of MCs, but clearly different from the fan-like, more restricted tuft described for classical eTCs (Pinching and Powell, 1971; Hayar et al., 2004b).

Although sTCs were described with both, bursting and non-bursting firing properties (Liu and Shipley, 1994; Kiyokage et al., 2010; Nagayama et al., 2014), according to Antal et al. (2006) the absence of bursting in juxtaglomerular TCs strongly predicts the presence of lateral dendrites, as found for all our VPCs. Conversely, in our sample of classical eTCs without lateral dendrites we were always able to reproduce bursting firing patterns and observed spontaneous bursts in more than half of the cells. Therefore, the observed lack of bursting in VPCs seems not to be related to our recording conditions. VPCs showed sags during long hyperpolarizing current injections (−100 to −120 mV), which are smaller in amplitude compared with sags recorded from our sample of eTCs at comparable hyperpolarization. These sags are typically mediated via hyperpolarization-activated currents (*I*_h_). Varieties of *I*_h_ channels were shown to be expressed in all subtypes of juxtaglomerular TCs, including eTCs and sTCs, with a higher prevalence for HCN4 in sTCs (Holderith et al., 2003; Fried et al., 2010). However, in our hands VPCs lack L-/T-type Ca^2+^ channel mediated LTSs during firing, a prerequisite for intrinsic spontaneous activity in bursting eTCs (Liu and Shipley, 2008) that we also recorded from eTCs during spontaneous bursts or the rebound phase following hyperpolarizing current steps. Additionally, the presence of LTSs is reflected in the very low last/first AHP ratio in the firing patterns of eTCs compared with the other cell types in our analysis, including VPCs. It should be mentioned, however, that the discrepancy between the Tobin et al. (2010) paper and our study with respect to the occurrence of bursts might be related to the young age of the rats in our dataset, because conductances relevant for bursting could be developmentally regulated (Kanyshkova et al., 2009). Then again, rats in the Antal et al. (2006) study were older than in ours, presumably overlapping with the Tobin et al. (2010) study. Further in line with Antal et al. (2006), another criterion to classify VPCs as non-bursting sTCs rather than eTCs is their slow membrane time constant (τ_m_) because we found VPCs to display a twofold slower τ_m_ than MCs and mTCs and even fourfold slower than eTCs.

Thus, the results from both neuroanatomical and electrophysiological characterizations suggest that VPCs correspond to the sTC subtype of TCs or a non-bursting subclass thereof. Interestingly, a recent study by Tavakoli et al. (2018) used cluster analysis of randomly patched juxtaglomerular cells in mice based on dendritic morphology and electrophysiological properties and identifies a previously unknown cluster E of “vertical superficial tufted cells”. Cluster E likely overlaps with VPCs because these cells feature a similar dendritic/axonal morphology, large somata (98.9 µm^2^), and similarly high τ_m_ (40.7 ± 20.1 ms) as well as R_i_ (0.65 ± 0.31 GΩ), and a low CV of ISI (0.17 ± 0.10). Tavakoli et al. (2018) also noted the similarity of cluster E with the type 2-sTCs described by Antal et al. (2006), whereas they propose VPCs to be part of their cluster G (“horizontal superficial tufted cells”; Antal et al., 2006 , their Table 5). Based on our observations and their characteristic vertical orientation of lateral dendrites and axons, we rather expect VPCs to be identical to cluster E or at least a subpopulation thereof. Intriguingly, Tavakoli et al. (2018) could not find synaptically connected pairs between other juxtaglomerular neurons and cluster E cells, which might be related to the tortuous apical dendrite and the overall low local excitatory connectivity observed here.

Although nothing is known on synaptic inputs and other network interactions of cluster E sTCs so far (Tavakoli et al., 2018), sTCs in general have been suggested to integrate feedback information of interneurons in the GL and EPL and even of GABAergic network inputs from superficial GC dendrites via both their pronounced dendritic tuft and lateral dendrites, whereas classical eTCs are obviously limited to input from the GL (Macrides and Schneider, 1982; Antal et al., 2006). Additionally, the strong dendritic innervation of the GL was suggested to imply that sTCs might be optimized to receive excitatory sensory signals (Antal et al., 2006), either via direct ON input or mediated via eTCs (De Saint Jan et al., 2009). However, this scenario is rather unlikely to hold for VPCs because under our recording conditions electrical ON stimulation primarily caused strong inhibition of VPCs, which occurred mostly via their tuft, whereas the lateral dendrites were not found to receive ON-mediated inputs.

### Possible origins of excitatory inputs to VPCs: sensory versus centrifugal

**Figure 9.**
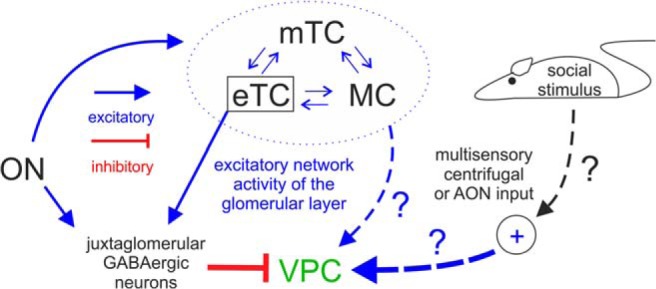
Graphic summary of detected inputs to olfactory bulb VPCs. Blue arrows represent excitatory inputs indicated by this study and from the literature. Red lines represent inhibitory inputs indicated by this study. The strength of the lines indicates the strength of the input. The cells within the dashed blue circle indicate the excitatory network within the same home glomerulus. Arrows labeled with question marks indicate speculative excitatory inputs to VPCs.

Because the glomerular synaptic connectivity of VPCs was not known and endogenous VP release is supposed to happen during presentation of volatile social odors (Lévy et al., 1995), we initially presumed that like classical eTCs, VPCs might receive excitation from the ON (Hayar et al., 2004b). As stated, to our knowledge it has not been investigated before whether vertical sTCs (cluster E) receive excitation directly from the ON and/or via eTCs, whereas horizontal sTCs were observed to receive inputs from classical eTCs (cluster G; Tavakoli et al., 2018). In our study ON stimulation does not result in immediate excitation but predominantly causes GABA_A_ receptor-mediated polysynaptic inhibition of VPCs as determined by the glutamatergic nature of ON transmitter release and the long latency (∼10 ms). Thus these inputs to VPCs might be generated either disynaptically via direct ON excitation of GABAergic interneurons or via the ON → eTC→ periglomerular cell circuit, like most GABAergic inhibition in the GL (Fig. 9; Aungst et al., 2003; Hayar et al., 2005). Finally, we also found that 50 Hz stimulation of the ON could not reverse VPC inhibition.

Although our findings imply that direct monosynaptic excitation of dendritic tufts of VPCs via the ON is unlikely to exist, the pharmacological blockade of the ON-evoked IPSPs unmasked barrages of depolarizing potentials that occurred with a yet longer latency than the IPSPs. Because tuftless VPCs never showed any excitatory responses to ON stimulation, these barrages may reflect excitatory local glomerular network reverberations between eTCs and projection neurons, i.e., MCs and mTCs (Fig. 9; De Saint Jan et al., 2009). Similar barrages on ON stimulation have been observed previously in MCs (“long-lasting depolarizations”; Aroniadou-Anderjaska et al., 1999; Carlson et al., 2000). This hypothesis is also supported by the very long and highly variable barrage onset latency (Nicoll, 1971). Still, it remains to be clarified whether these excitatory inputs to VPCs are originating from MC/mTCs and/or eTCs and/or else.

Thus to excite VPCs, inputs are required that either inhibit the GABAergic origin of the ON-evoked inhibition (i.e., disinhibition) and/or deliver enough direct excitation to outweigh the inhibition. These additional inputs could restrict bulbar VP release to occasions when social odors are processed. For example, the detection of pheromones in the AOB could provide the required specificity for social stimuli via local excitatory inputs to the main OB (Vargas-Barroso et al., 2016). Another candidate region for social-specific inputs is the anterior olfactory nucleus (AON), which provides numerous glutamatergic centrifugal afferents to the OB (Markopoulos et al., 2012; Rothermel and Wachowiak, 2014) and receives projections from the hypothalamus, that enhance input from the AON to OB granule cells during social interactions, resulting in an improved signal-to-noise ratio of olfactory input processing (Oettl et al., 2016). A similar social interaction-driven excitation of VPCs via AON projections to the GL seems plausible (Luskin and Price, 1983). Finally, the perception of other, non-olfactory sensory social cues (visual, auditory, tactile) could act as top-down social go-signal (Fig. 9). The most prominent modulatory centrifugal inputs that could mediate such signals include noradrenergic fibers from the locus ceruleus, cholinergic fibers from the horizontal limb of the diagonal band of Broca and serotonergic fibers from the dorsal raphe nucleus (Matsutani and Yamamoto, 2008), because all three neuromodulatory systems were shown to be involved in facilitating social odor discrimination (Lévy et al., 1995; Dluzen et al., 1998a; Cavalcante et al., 2017).

### Mechanisms of dendritic VP release in OB versus hypothalamic VPCs

Although so far the mechanisms for suprathreshold VPC excitation and thus subsequent release of VP are not yet known, several of our findings and previous observations suggest that VPCs are able to release VP within the cellular network of the OB from both dendrites and axons:

 (1)The observed VP immunoreactivity in soma, dendrites, and axons indicates that these structures are potential release sites. Unfortunately, because of the low immunofluorescence of VP-neurophysin we could not prove that VP is present also within the finer branches of the neurites. Yet, early histological studies by De Vries et al. (1985) describe “scattered elongated” VP-immunoreactive fibers in the EPL of the rat OB. Because we observed that VPC axons are widely spread throughout the EPL, we would like to suggest that all VPC substructures express VP.

 (2)The presence of VP-receptive VP and oxytocin receptors (Manning et al., 2012) throughout all layers of the OB (Vaccari et al., 1998; Tobin et al., 2010) indicates that several components of the OB cellular network are able to detect endogenous VP release.

 (3)The observation of effects of exogenous VP application on ON-induced synaptic inputs to two OB cell types with glomerular dendritic tufts (sTCs/VPCs and eTCs), indicates a functional relevance of VP signaling in olfactory processing. This notion is strongly supported by earlier findings demonstrating that blockade of endogenous VP receptors via intrabulbar infusion of a selective VP receptor antagonist reduces MC excitation as well as social odor discrimination abilities *in vivo* (Tobin et al., 2010).

 (4)The occurrence of moderate Ca^2+^ entry into VPC apical tufts following somatic AP trains indicates the presence of voltage-gated Ca^2+^ channels (VGCCs) that could contribute to triggering VP release.

 (5)The threshold for AP generation in VPCs is similar to other TCs, like MCs and eTCs. Further, VPCs fire APs on both small positive current injections and the rebound following hyperpolarization. Thus, given that an adequate excitatory stimulus is present, VPCs should be sufficiently excitable to sustain AP trains that might be required for both dendritic and axonal release of VP.

Although the exact release mechanisms of VP from OB VPCs remain to be elucidated in future studies, a comparison of our findings with the release mechanisms of hypothalamic VPCs may also yield insights into this matter in bulbar VPCs. Hypothalamic VPCs release VP from axon terminals in the periphery, but also centrally from their dendrites and the surface of their soma (Pow and Morris, 1989). With respect to dendritic/somatic release mechanisms in general, MCs, GCs, and other dendritically-releasing neurons in the OB and elsewhere dispose of an effective dendritic AP backpropagation mediated by active dendritic conductances such as voltage-gated Na^+^ and Ca^2+^ channels (Stuart et al., 1997; Egger et al., 2003; Zhou et al., 2006). However, in VPCs we observed no Ca^2+^ entry on single backpropagating APs and only moderate intracellular Ca^2+^ transients in response to prolonged AP trains. These observations possibly indicate that substantial Ca^2+^ entry into VPC apical tufts sufficient for release cannot be achieved via somatic AP firing alone. In line with that idea, in hypothalamic VPCs antidromic axonal electrical stimulation (50 Hz for 3 s) is not enough to induce somatodendritic VP release (Ludwig et al., 2005), although dendritic Ca^2+^ spike propagation via VGCCs is possible during long-lasting current application (>400 ms; Bains and Ferguson, 1999). In hypothalamic VPCs dendritic release can be transiently uncoupled from peripheral axonal release in the neural lobe of the pituitary (Ludwig et al., 1994). Accordingly, Bains and Ferguson (1999) suggest that dendritic VGCCs (L-, N-, and T-type according to Sabatier et al., 1997) are located in some distance from the soma. Intriguingly, whereas the somata of OB VPCs are mostly located very superficially in the EPL, they always keep a certain distance from the glomerulus containing the tuft (100 µm), resulting in a longer apical dendrite below the tuft compared with the almost inexistent apical dendrite of classical eTCs (Pinching and Powell, 1971). Thus, the long VPC apical dendrite with its poor backpropagation may allow for a certain degree of functional compartmentalization, i.e., uncoupling between the tuft and the soma, which was also proposed as explanation for the long primary dendrites of MCs and TCs (Chen et al., 2002; Migliore et al., 2005). One possibility to induce somatodendritic VP release in the hypothalamus is the application of VP itself (Ludwig et al., 1994). In line with that finding, our experiments demonstrate that exogenous VP reduces ON-induced VPC inhibition. However, a direct excitatory effect of VP on VPCs is not supported by our results. Another known trigger for dendritic vesicle release in hypothalamic VPCs is postsynaptic Ca^2+^ influx via NMDA receptors (De Kock et al., 2004), and accordingly photolysis of caged-NMDA efficiently evokes dendritic VP release (Son et al., 2013). It is tempting to speculate that similar mechanisms also exist in the OB.

In this case a strong excitatory synaptic input to the apical dendrite would be required to enable VP release, thus the question remains where such synaptic inputs might originate from if not the ON?

### Possible targets of axonal VPC output and implications for social odor processing

Provided that during social odor sensing *in vivo* there are adequate inputs to activate the OB VP system, what would be the targets of axonal VP release in the OB? The more common morphological VPC type 1 densely innervates the EPL with numerous short branches that feature multiple but localized projections to the MCL and superficial GCL, possibly within a distinct functional modular column determined by its home glomerulus (Willhite et al., 2006). The second, less numerous type 2 also innervates the EPL but has long-ranging projections below the MCL reaching either medially into the GCL or along the internal plexiform layer. Projection patterns similar to that of VPC type 2 shown here were described for cholecystokinin (CCK) immunoreactive sTCs in the OB (Liu and Shipley, 1994; Ma et al., 2013). As CCK immunoreactivity is found in most subtypes of sTCs (Fried et al., 2010), type 2 VPCs in the OB may be a subpopulation of CCK cells. Interestingly, CCK cells were shown to be part of the intrabulbar association system, because axonal projections of CCK cells synapse onto GCs and MCs of the isofunctional glomerulus that receives inputs from the same olfactory receptor. This association results in a positive feedback circuit for amplifying glomerular outputs of the same stimulus (Liu and Shipley, 1994; Ma et al., 2013). If the type 2 VPCs were also part of the intrabulbar association system (which we could not demonstrate in acute slices), this would be an efficient way to globally amplify relevant social signals and thereby sharpen the profile of an individual social odor signature.

In the hippocampus, a brain region that relies on endogenous VP release to facilitate social odor discrimination in rodents, VP signaling generally increases GABAergic inhibition (Cilz et al., 2018). The dense axonal and dendritic innervation of type 1 and type 2 VPCs in the GL, EPL, and GCL would enable these processes to release VP onto GABAergic periglomerular cell and GC somata and their presynaptic dendrites. As VP receptors were shown to be expressed in both, GL and GCL (Vaccari et al., 1998) and application of VP inhibited eTC EPSPs *in vitro* as well as MC activity *in vivo* (Tobin et al., 2010), it is conceivable that VP-induced increased synaptic inhibition improves the discrimination of very similar odors, as known for nonsocial binary odor mixtures (Abraham et al., 2010). Indeed, an highly sensitive discrimination of social odors may be desirable as gas chromatography has revealed that the volatile component of individuals’ body odors contains largely overlapping sets of odor molecules and thus the individual identity is mainly coded via the relative composition of shared volatile components (Singer et al., 1997; Schaefer et al., 2002).

## Summary

VPCs are non-bursting sTCs that feature a subtype that seems to be predestined for involvement in local “glomerular”/columnar processing and one subtype that has the potential to be involved in more “global”, long-range intrabulbar network processing. Further, VPCs receive indirect excitatory and inhibitory inputs via the ON that are dominated by GABAergic signaling. Because we observed that ON inputs could not directly excite VPCs, the activation of the bulbar VP system possibly relies on additional direct or indirect modulatory inputs from within the olfactory system or upstream, multisensory pathways that are triggered by social stimuli. As to the output of VPCs, previous studies and our preliminary results indicate that VP is rather involved in increasing the inhibitory signaling in the OB.

## Synthesis

Reviewing Editor: Anna Menini, SISSA (International School of Advances Studies) Decisions are customarily a result of the Reviewing Editor and the peer reviewers coming together and discussing their recommendations until a consensus is reached. When revisions are invited, a fact-based synthesis statement explaining their decision and outlining what is needed to prepare a revision will be listed below. The following reviewer(s) agreed to reveal their identity: Michele Dibattista.

This manuscript characterizes structurally and electrophysiologically a class of cells in olfactory bulb, those being the Vasopressin cells (VPCs). The detailed analysis of neuro-anatomical structure and electrical properties of VPCs represents the first step to understand how vasopressin system work in the olfactory bulb circuitry. The revised manuscript is much improved. The abstract and significance statements match the results described, without reducing the impact. The additional experiments have solidified some of the initial observations, strengthening authors' interpretation, and as a result, the entire study.

A minor point still needs to be addressed: the graphical summary in Figure 9 is useful, but the nature of excitatory input to the VPC is speculative at best. Authors should put question marks next to the arrows. These will distinguish what is solid (and shown in this manuscript) and what is not, and would portray a more accurate picture to the readers.

## Author Response

Synthesis Statement for Author (Required):

This manuscript characterizes structurally and electrophysiologically a class of cells in olfactory bulb, those being the Vasopressin cells (VPCs). The detailed analysis of neuro-anatomical structure and electrical properties of VPCs represents the first step to understand how vasopressin system work in the olfactory bulb circuitry. The revised manuscript is much improved. The abstract and significance statements match the results described, without reducing the impact. The additional experiments have solidified some of the initial observations, strengthening authors' interpretation, and as a result, the entire study.

A minor point still needs to be addressed: the graphical summary in Figure 9 is useful, but the nature of excitatory input to the VPC is speculative at best. Authors should put question marks next to the arrows. These will distinguish what is solid (and shown in this manuscript) and what is not, and would portray a more accurate picture to the readers.

Reply: As advised by the editor we no inserted question marks next to the excitatory inputs to the VPCs to indicate their speculative nature. We also mention that now in the figure legend.
